# Foldable and Cytocompatible Sol-gel TiO_2_ Photonics

**DOI:** 10.1038/srep13832

**Published:** 2015-09-07

**Authors:** Lan Li, Ping Zhang, Wei-Ming Wang, Hongtao Lin, Aidan B. Zerdoum, Sarah J. Geiger, Yangchen Liu, Nicholas Xiao, Yi Zou, Okechukwu Ogbuu, Qingyang Du, Xinqiao Jia, Jingjing Li, Juejun Hu

**Affiliations:** 1 University of Delaware, Department of Materials Science & Engineering, Newark, Delaware 19716, USA; 2 Tianjin University, School of Electronic and Information Engineering, Tianjin 300072, China; 3 University of Hawaii at Manoa, Department of Mechanical Engineering, Honolulu, Hawaii 96822, USA; 4 University of Delaware, Biomedical Engineering Program, Newark, Delaware 19716, USA; 5 Massachusetts Institute of Technology, Department of Materials Science & Engineering, Cambridge, Massachusetts 02139, USA

## Abstract

Integrated photonics provides a miniaturized and potentially implantable platform to manipulate and enhance the interactions between light and biological molecules or tissues in *in-vitro* and *in-vivo* settings, and is thus being increasingly adopted in a wide cross-section of biomedical applications ranging from disease diagnosis to optogenetic neuromodulation. However, the mechanical rigidity of substrates traditionally used for photonic integration is fundamentally incompatible with soft biological tissues. Cytotoxicity of materials and chemicals used in photonic device processing imposes another constraint towards these biophotonic applications. Here we present thin film TiO_2_ as a viable material for biocompatible and flexible integrated photonics. Amorphous TiO_2_ films were deposited using a low temperature (<250 °C) sol-gel process fully compatible with monolithic integration on plastic substrates. High-index-contrast flexible optical waveguides and resonators were fabricated using the sol-gel TiO_2_ material, and resonator quality factors up to 20,000 were measured. Following a multi-neutral-axis mechanical design, these devices exhibit remarkable mechanical flexibility, and can sustain repeated folding without compromising their optical performance. Finally, we validated the low cytotoxicity of the sol-gel TiO_2_ devices through *in-vitro* cell culture tests. These results demonstrate the potential of sol-gel TiO_2_ as a promising material platform for novel biophotonic devices.

Besides its well-established roles in communications and information technology, photonics is increasingly penetrating into other emerging application arenas, in particular, biotechnology and healthcare^1^. Integrated photonic devices are uniquely poised for *in-vitro* and *in-vivo* sensing, diagnostics, therapeutics, and stimulation functions given their small form factor, low power consumption, robustness, large multiplexing capacity, as well as strong light-molecule/tissue interactions enabled by tight optical confinement in these devices. Nevertheless, conventional photonic integration is based on rigid substrate platforms such as semiconductors or glass, and their mechanical stiffness makes the resulting devices inherently incompatible with soft biological tissues. For instance, the large elastic mismatch between optogenetic neural probes and the brain tissue contributes to undesired tissue reactions such as glial scarring and tissue encapsulation^2^. Conformal sensor integration on human skin serves as another example where device mechanical flexibility becomes indispensable. In addition to mechanical stiffness, the presence of residual chemicals used in the photonic device processing, which may be toxic, can present another potential barrier towards biophotonic applications^3^.

Traditional solutions to the aforementioned challenges entail exclusively using compliant, biocompatible organic polymeric materials for photonic device fabrication. A few examples of such materials include silk fibroin, gelatin, and agarose hydrogel^4^,^5^,^6^. The limited material selections and the low index contrast available in organic systems, however, pose severe constraints on the photonic functionalities that can be attained in these materials. Hybrid inorganic-organic photonics, exemplified by nanomembrane-based devices^7^,^8^,^9^,^10^,^11^,^12^, offers a preferred solution for flexible photonic integration given the diverse repertoire of photonic components already demonstrated based on these systems. In the same vein, our group recently demonstrated the superior optical and mechanical performance of our hybrid inorganic-organic flexible photonic devices based on chalcogenide glass materials^13^,^14^,^15^. However, the biocompatibility of the constituent materials and residual chemicals used in processing these hybrid photonic devices, despite its critical importance to many *in-vitro* and *in-vivo* applications, has rarely been addressed^16^,^17^,^18^.

In this article, we investigate amorphous TiO_2_ thin films deposited using a low-temperature sol-gel process as a new material for integrated biophotonic components. TiO_2_ is an ideal material for integrated biophotonics for several reasons. First, it can be deposited and processed in a monolithic manner to form thin film devices at reduced temperatures compatible with flexible substrate integration. Second, TiO_2_ exhibits a broad optical transparency window stretching from approximately 400 nm to 5.5 μm in wavelength^19^, which covers most important wave bands for biophotonic applications including fluorescence imaging, optogenetic excitation, Raman and infrared spectroscopy. Third, TiO_2_ is known as a biocompatible material, which justifies their use in cosmetic products^20^, dental fillers^21^,^22^ and artificial bones^22^ (it is, however, worth noting that while TiO_2_ itself is biocompatible, chemicals or processing steps involved in deposition and microfabrication of TiO_2_ devices may introduce toxic molecules). Forth, the material is no stranger to the silicon microelectronics industry, as it has been employed as a high-k dielectric material^23^ which qualifies it as CMOS-compatible and potentially opens up opportunities to leverage existing foundry facilities and knowledge base in device processing. Finally, TiO_2_ exhibits superior chemical and thermal stability essential for multi-step microfabrication.

While TiO_2_ thin films have been widely used in photocatalysis, dye-sensitized solar cells, and as anti-reflective and/or antibacterial coatings^24^,^25^,^26^,^27^, their application in integrated photonics has only been explored in a few recent reports. Zhang *et al.* fabricated a high sensitivity photonic crystal biosensor with TiO_2_ nanorod structure^28^,^29^. Furuhashi *et al.* showed a propagation loss of 9.7 dB/cm at 632 nm wavelength for 10 μm wide TiO_2_ waveguides made of reactive sputtered films. The authors suggested that the present loss was mainly due to partial crystallization of the sputtered films^30^. Hayrinen *et al.* used atomic layer deposition (ALD) to fabricate TiO_2_ waveguides and reported a loss of 5.0 dB/cm at 1550 nm wavelength^31^. Bradley *et al.* and Choy *et al.* demonstrated waveguides and micro-ring resonators made of reactive sputtered TiO_2_ films in both amorphous and polycrystalline (anatase) states, and they achieved a Q-factor of 22,000 near 632 nm wavelength^32^. More recently, Park *et al.* reported fabrication of whispering gallery mode (WGM) resonators in sol-gel TiO_2_ by HF wet etch and claimed a high Q-factor of 10^5^ at 980 nm wavelength^33^. In general, amorphous TiO_2_ films exhibit lower loss than their polycrystalline counterpart. For sputtered films, the propagation loss reduces at longer wavelengths, which indicates that scattering is the likely loss mechanism. On the other hand, loss reaches a minimum at 980 nm wavelength in sol-gel films and rises to 15 dB/cm at 1550 nm, possibly suggesting the presence of residual organics that cause optical attenuation at longer wavelengths. In the prior reports the devices are exclusively integrated on silicon or glass substrates.

Here, we choose to pursue a sol-gel synthetic route free of organic species to enable flexible substrate integration since TiO_2_ films deposited by traditional evaporation or sputtering processes are not suitable for photonic integration on flexible substrates. TiO_2_ films evaporated in vacuum or sputtering in a pure argon ambient are oxygen deficient and therefore exhibit high optical losses not suitable for guided wave photonic applications. The oxygen loss issue can be mitigated by plasma-assisted reactive deposition in an oxygen environment, although the process leads to severe thermal and plasma damage to polymer substrates due to a combined effect of plasma ashing and the Ti+O_2_→TiO_2_ exothermic reaction (∆H = −945 kJ/mol), a unique challenge associated with flexible substrate integration. The conclusions are supported by our own experimental results on sputtered deposited TiO_2_ films presented in the Additional Information. Structural and optical properties of sol-gel films were systematically characterized to define the optimal film deposition conditions with minimal optical loss. Single-mode waveguides and resonator devices were patterned on flexible substrates using reactive ion etching (RIE), and their mechanical and optical performance was tested and quantitatively accounted for using finite element mechanical modeling. We further performed cell culture experiments to confirm the cytocompatibility of the flexible TiO_2_ devices.

## Results and Discussion

### TiO_2_ thin film synthesis, device fabrication and optical characterizations

**Figure 1:**
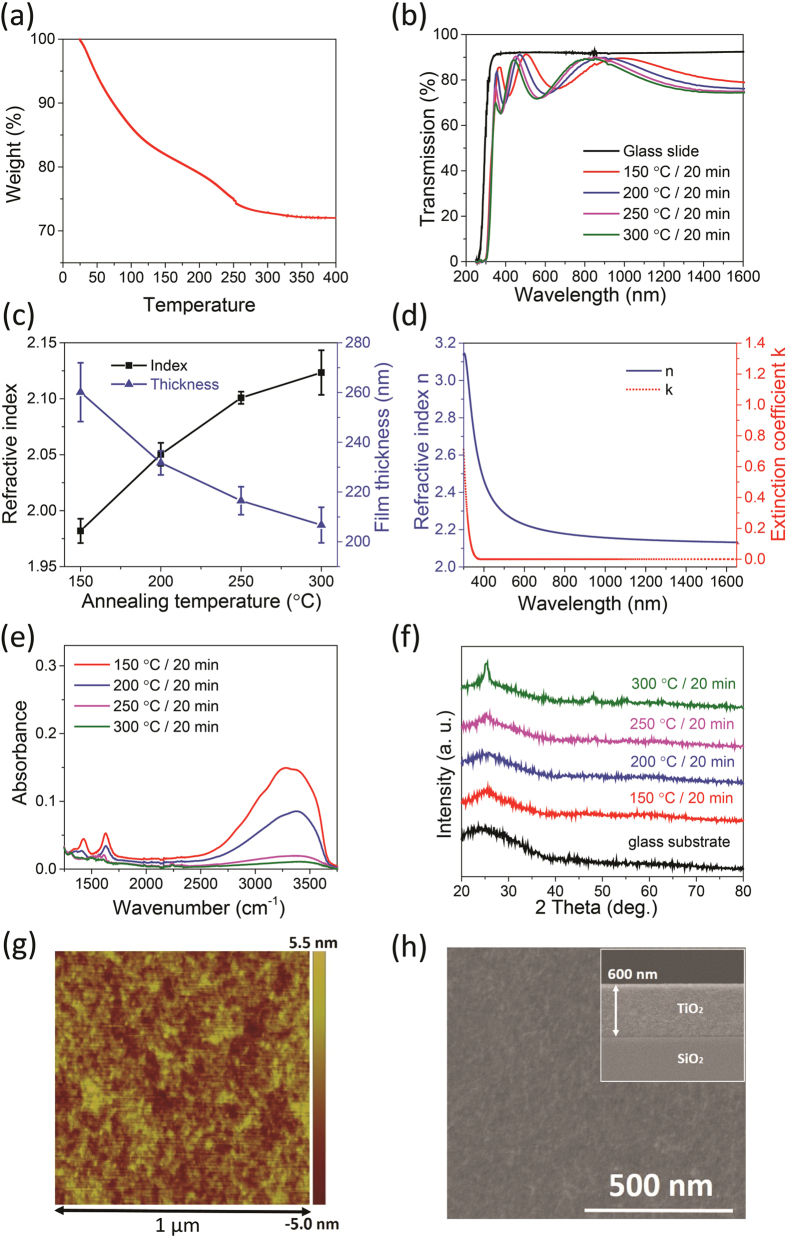
Characterization of sol-gel prepared TiO_2_ thin films annealed at different temperatures. (**a**) thermogravimetric analysis (TGA) curve of pre-dried PTA sol; (**b**) UV-Vis transmission spectra of the films on glass substrates; (**c**) refractive indices at 1550 nm wavelength and film thickness, both fitted from the UV-Vis spectra using the Swanepoel method; (**d**) refractive indices n and extinction coefficients k of TiO_2_ thin film annealed at 250 °C measured using ellipsometry; (**e**) FTIR spectra; (**f**) X-ray diffraction spectra; (**g**) AFM surface profile (1 μm × 1 μm); (**h**) top-view SEM image of film annealed at 250 °C; inset: film cross-section.

TiO_2_ sol was prepared using the sol-gel process described in the supporting information. During film deposition, 0.4 mol/L peroxo titanic acid (PTA) sol was spin-coated on soda-lime glass or silicon substrates at a spin speed of 1500 rpm for 0.5 seconds. The spin process was repeated 3 times to obtain a target film thickness of 380 nm. Between the spin cycles, the film was baked at 150 °C for one minute to remove residual water in the layer deposited in the previous cycle. Post-deposition annealing followed after the entire coating process to stabilize the resulting film structure. To identify the optimal annealing condition, thermogravimetric analysis (TGA) was performed on the pre-dried PTA sol (baked in vacuum oven at 80 °C for 24 hours). TGA results shown in Fig. 1a suggest that the weight loss of PTA sol occurs primarily from 50 °C to 250 °C, which is attributed to loss of coordinated water and decomposition of peroxo groups. The conclusion is further supported by optical characterizations of the films. Figure 1b shows the UV-V is transmittance spectra of TiO_2_ thin film annealed at different temperatures. Refractive indices and thicknesses of the films were calculated from the spectra using the Swanepoel method^34^ and plotted in Fig. 1c. The fitted indices also agree well with our ellipsometry measurements (Fig. 1d), showing the index and absorption dispersion of film annealed at 250 °C. As the annealing temperature increases, removal of water and NH_3_ leads to denser film microstructures, which accounts for the film thickness reduction and index increase. Figure 1e plots the Fourier Transform InfraRed (FTIR) absorption spectra of the films. The peaks located at 1420 cm^−1^ and 1620 cm^−1^ are attributed to N-H stretching and -OH bending vibration, respectively^35^, and the broad absorption band from 3000 cm^−1^ to 3600 cm^−1^ is assigned to the stretching vibration of the hydrogen-bonded OH groups of the adsorbed water^35^. All absorption peaks progressively diminish as the annealing temperature increases, with the most significant peak height reduction occurring between 150 °C to 250 °C. While raising the annealing temperature to 300 °C does contribute to further removal of -OH and -NH species, X-ray diffraction analysis shows partial crystallization in films annealed at 300 °C, evidenced by the anatase phase [101] diffraction peak at 26° (Fig. 1f). Therefore, we choose to a post-deposition annealing temperature of 250 °C based on these results. TiO_2_ films prepared under this optimal annealing condition also exhibit a dense, defect-free microstructure and a low surface RMS roughness of (1.2 ± 0.2) nm confirmed by atomic force microscopy (AFM) surface morphology measurement (Fig. 1g) and SEM cross-sectional imaging (Fig. 1h). The high optical and structural quality of the films facilitates low-loss photonic device fabrication.

**Figure 2:**
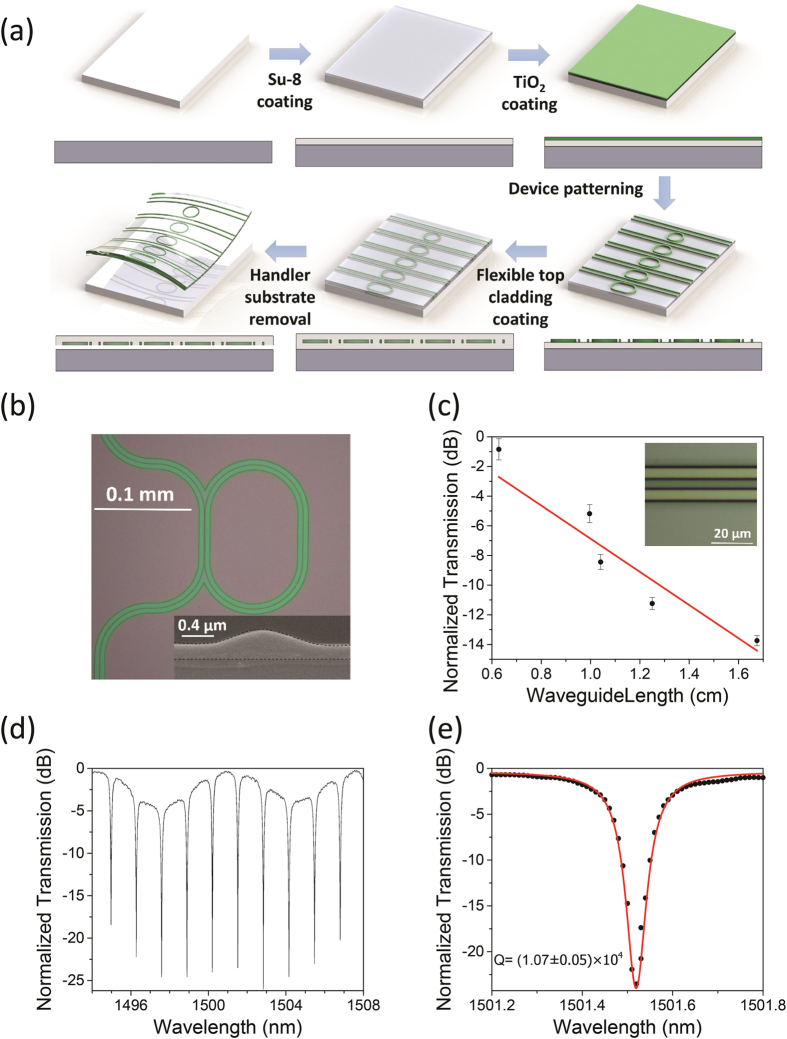
Flexible TiO_2_ photonic device fabrication and optical characterization. (**a**) Schematic device fabrication process; (**b**) optical microscope top-view image of a TiO_2_ rib racetrack resonator. The inset shows the cross-sectional SEM image of the bus waveguide; (**c**) cut-back loss measurement: transmitted optical power as a function of waveguide length at 1550 nm wavelength. The channel waveguide width is 4.7 μm and height is 0.2 μm. Inset shows an optical microscope image of a fabricated TiO_2_ channel waveguide; (**d,e**) normalized optical transmission spectra of a TiO_2_ rib racetrack resonator with a loaded Q-factor of (1.07 ± 0.05) × 10^4^.

We fabricated TiO_2_ photonic devices following protocols illustrated in Fig. 2a, where we leverage established planar microfabrication technologies to monolithically integrate devices on unconventional flexible substrates. In the process, an oxidized silicon wafer serves as a handler substrate to provide mechanical support during device processing. The wafer is coated with a layer of SU-8 epoxy, on which the TiO_2_ films are deposited using the sol-gel method described in the preceding section and patterned into photonic structures. The lithography was performed on an i-line mask aligner with a negative photoresist (NR9-1000PY, Futurrex). The resist pattern was reflowed at 135 °C for 4 s on a hot plate after development to remove line edge roughness. Device patterns were transferred to the TiO_2_ layer using Inductively Coupled Plasma (ICP) RIE in mixed CF_4_, Ar and O_2_ gases (volume ratio 16:4:3, total pressure 2 Pa) and at a microwave power of 500 W with 150 W bias. The etch rates of the resist and TiO_2_ are 300 nm/min and 100 nm/min under the etching condition, respectively. This low etch selectivity, although sufficient for defining our rib waveguide geometry (Fig. 2b), can be improved by using a hard mask when deep etch is required^36^. After etching, each the sample was sonicated in acetone to remove the remaining resist mask followed by another layer of SU-8 top cladding coating. Finally, the sample was cleaved along with the handler substrate to prepare waveguide facets and subsequently delaminated from the handler substrate using a Kapton tape to form free-standing flexible membrane devices. Figure 2b shows a top-view optical micrograph and an SEM cross-sectional image of a fabricated racetrack resonator. RMS surface roughness on the waveguides was quantified using AFM to be (1.4 ± 0.3) nm (Figure S2a) prior to top SU-8 cladding coating. However, we also found scattered resist residues along the waveguides (Figure S2b), which likely account for the observed scattering loss.

The flexible TiO_2_ waveguides and resonators were mounted on motion stages and characterized using the fiber end fire coupling method *in-situ* both in a “flat”, undeformed state and when subjected to bending. The testing protocols are similar to what we used to characterize chalcogenide glass based flexible photonic devices and are detailed in the Supporting Information as well as our previous publication^15^. Propagation loss of the buried flat channel waveguides near 1550 nm wavelength was measured using the standard cut back method prior to delamination from the handler substrate. Insertion loss of waveguides of different lengths is plotted in Fig. 2c, and the propagation loss is inferred from the slope to be (11 ± 2) dB/cm. Figure 2d and Fig. 2e present a typical transmission spectrum of a flexible TiO_2_ resonator. Using the coupled mode theory, an intrinsic Q-factor of 2 × 10^4^ is fitted from the spectrum. The loss number and Q-factor are comparable to previously reported values in sol-gel devices: Park *et al.* measured a propagation loss of 15 dB/cm and an intrinsic Q-factor of 2 × 10^4^ in TiO_2_ WGM resonators near 1550 nm wavelength^33^. The losses result from both intrinsic and extrinsic contributions. Intrinsic losses are due to the absorption of NH and OH vibrations overtones in the NIR region and the Rayleigh scattering of possible micro-voids remaining in the sol-gel TiO_2_ thin films^37^. Intrinsic material loss in the films was evaluated to be (3 ± 1) dB/cm at 1550 nm wavelength by slab mode propagation loss measurement on a Metricon 2010 prism coupler with a fiber bundle attachment. Therefore, extrinsic losses originating from scattering by discrete defects such as resist residue account for a large fraction of the observed waveguide loss, which can be mitigated through further lithography and etching process optimization.

### Mechanical modeling and testing

We have recently pioneered a multi-neutral-axis configurational design that enables robust flexible photonic devices^15^,^38^. This novel configuration leads to the emergence of multiple neutral axes in the laminates where the strains vanish. If photonic components are placed at these neutral axes then they can sustain large mechanical deformation without breaking or degradation. By adjusting the modulus and thickness of the layers, the locations of the neutral axes can be flexibly tuned across the stack to meet diverse application needs, a major advantage of our approach. Here we extend the basic design principle to demonstrate foldable photonics that can sustain repeated bending with extremely small radius (*R* = 0.25 mm) on substrates approximately 0.1 mm thick. However, rather than using the multi-neutral-axis analytical theory we developed to model our devices, we resort to the finite element method (FEM) to compute strain distribution during mechanical deformation of the devices for two reasons. First, the extraordinary mechanical flexibility of our devices demonstrated here enables large deformation (*R* is only 2.5 times of *d*), and the assumptions underlying our prior analytical model do not necessarily hold in this large deformation regime. Second, the multi-neutral-axis theory does not take into account the embedded photonic device layer in the analysis. This assumption is valid when the devices consist of channel waveguides as shown in our previous report; nevertheless, rib waveguide structures introduce a continuous TiO_2_ slab layer with much higher modulus compared to the surrounding polymers, which warrants further inspection of the analytical model.

**Figure 3:**
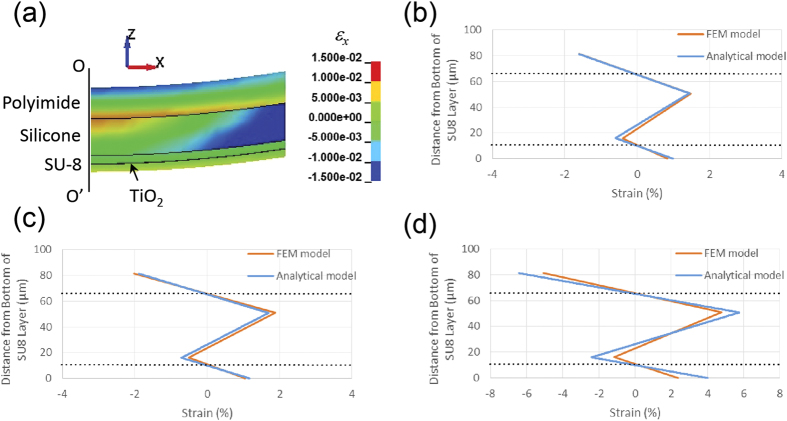
Mechanical simulation. (**a**) Strain distribution in the laminated photonic chip structure during bending at R = 1 mm; (**b–d**) strain *ε*_*x*_ along the structure’s center axis OO’ calculated using FEM and the analytical multi-neutral-axis model: (**b**) R = 1 mm, (**c**) R = 0.85 mm, and (**d**) R = 0.25 mm. The black dotted lines mark the locations of the neutral axes in the polyimide and SU-8 layers.

The FEM model was created using the LS-DYNA solver. Details of the FEM model settings and the impact of the TiO_2_ layer on strain distribution are discussed in the Supporting Information. Figure 3a plots the distribution of strain component *ε*_*x*_ when the flexible photonic chip is bent simulated by FEM, and Fig. 3b–d compare the strain component *ε*_*x*_ along the center axis OO’ calculated using FEM and the multi-neutral-axis analytical model^15^ under bending radii of 1 mm, 0.85 mm, and 0.25 mm, respectively. For the relatively large bending radii (*R*) of 1 mm and 0.85 mm, the results from FEM and analytical methods are consistent. However, at *R* = 0.25 mm, the analytical model over-predicted the strain by up to 100% since the thickness reduction of each layer was not considered in the analytical model. Despite the large deviation of strain, we note that the analytical model still captures two critical features: strain distribution in the SU-8, polyimide and silicone layers remains linear across the thickness direction, an assumption of the multi-neutral-axis theory; and, more importantly, the laminated structures still exhibit neutral axes in the SU-8 and polyimide layers at locations coinciding with the analytical model predictions and independent of the bending radius. This interesting observation suggests that while our multi-neutral-axis theory overestimates strains inside a laminate under large deformation, it can still be practically applied to design compliant photonic and electronic devices with extraordinary flexibility by placing the device layer at the model-predicted neutral axis location, since the zero strain condition at the device layer is not compromised even under large deformation.

**Figure 4:**
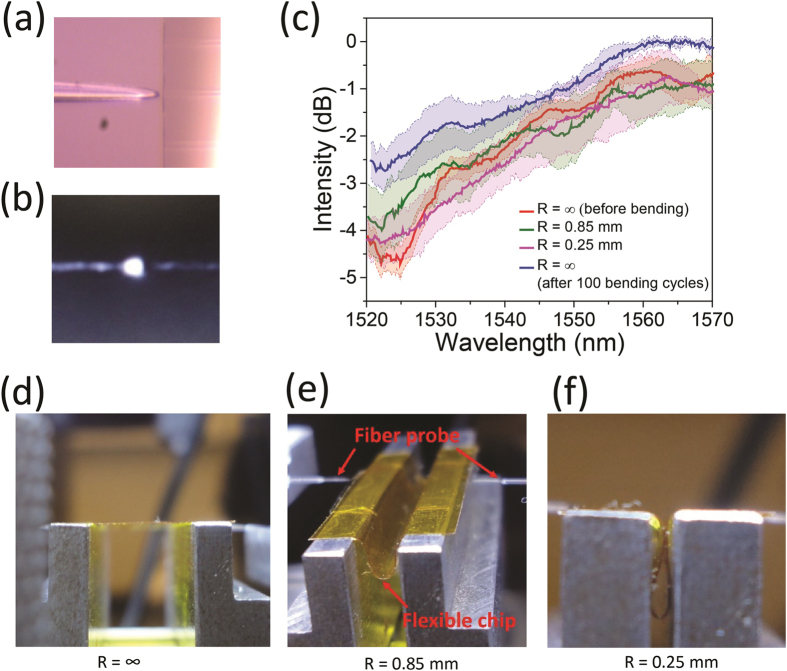
Mechanical tests of foldable TiO_2_ waveguides. (**a**) optical microscope image of the input fiber coupled to a waveguide; (**b**) far-field image of TE polarized mode output from a flexible TiO_2_ waveguide; (**c**) normalized optical transmission spectra of a flexible waveguide after bending at different radii; (**d–f**) photos of the fiber butt coupling testing set-up for *in-situ* measurement of optical transmission characteristics at different bending radii.

Based on the modeling results, we fabricated flexible TiO_2_ photonic devices by aligning the device layer with the model-predicted neutral axis locations. Figure 4a shows an optical image of the fiber coupled to an end facet of the flexible waveguides and Fig. 4b presents a far-field image of the TE mode output from the waveguide. Figure 4c shows the transmission spectra of a flexible TiO_2_ waveguide following the multi-neutral-axis configuration design. The solid lines correspond to average transmittance through the waveguide when it is undeformed, bent to different radii, and after 100 bending cycles at 0.25 mm radius. The shaded regions denote the standard deviation of waveguide transmittance due to coupling variation. The different colored shaded regions overlap, indicating minimal optical loss variation of the devices after the bending cycles. Structural integrity of the devices after bending was also confirmed via optical microscope inspection, and no cracks or delamination of the layers were observed. The result represents the best-in-class performance for flexible waveguide-based devices.

### Cytotoxicity testing

**Figure 5:**
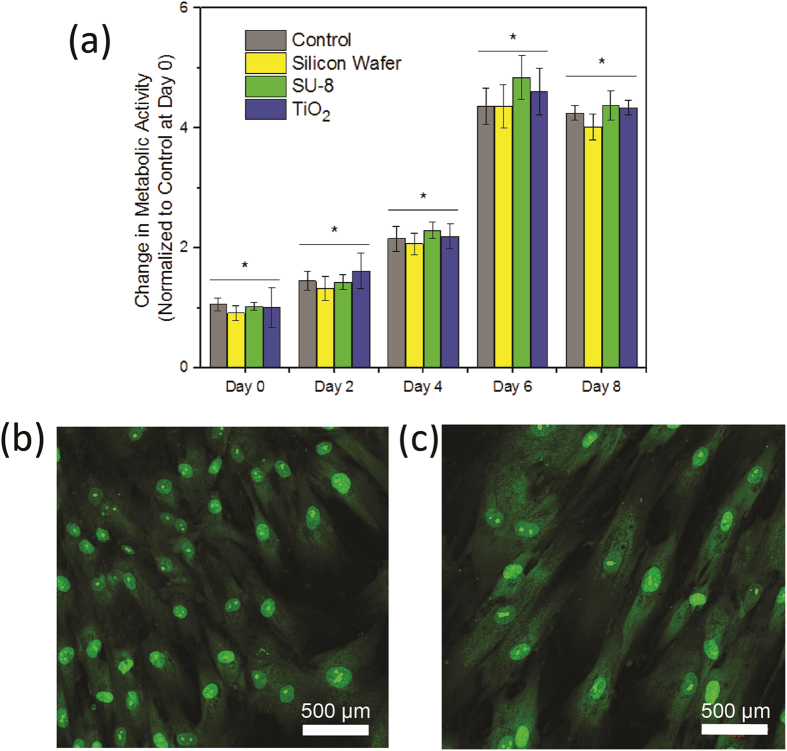
Analyses of cytocompatibility of the sensor materials. (**a**) proliferation of hMSCs in indirect contact with sensor materials; (**b–c**) confocal images of live/dead stained day 10 hMSCs cultured in direct contact with SU-8 (**b**); and TiO_2_ (**c**). Live cells were stained green and dead cells, if any, were stained red. *significantly different (*p* < 0.01) from day 0–6. No significant difference was observed between day 6 and 8.

To assess the cytocompatibility of our sensor materials (SU-8 and TiO_2_), we cultured human mesenchymal stem cells (hMSCs) in proximity to and in direct contact with thin layers of each sensor material following established cell culture procedures^39^,^40^. Thin films of TiO_2_ were deposited on bare silicon wafer via the sol-gel process. SU-8 2002 photoresist was spin coated on select samples to simulate sensor encapsulation in SU-8. The samples were then cleaved and suspended in cell culture inserts above hMSCs cultured in a 12-well plate. Bare silicon wafer was also included in the study as an additional test substrate. Three chips of each sample were included in the study, and three empty trans-well membranes were included in control wells. As shown in Fig. 5a, hMSCs proliferated over six days of the study, after which the cell monolayer became confluent, prohibiting further cell growth (Figure S3 in Supporting Information). On each day, no statistically significant difference was found between test samples and control groups. The metabolic activity of hMSCs increased significantly (*p* < 0.01) after the culture was initiated until cells reached confluence. By day 8, the average number of cells in all testing groups increased by approximately four folds from day 0, confirming that the SU-8 and TiO_2_ films did not compromise the proliferative potential of hMSCs. When plated directly on the sensor materials, hMSCs attached, adopted a spindle-shaped morphology and maintained a high viability (Fig. 5b–c) with little to no cell death. Collectively, our results confirmed the cytocompatibility of our sensor materials.

## Conclusion

In this study, we demonstrated foldable and cytocompatible photonic devices based on sol-gel synthesized TiO_2_ thin films. Our FEM mechanical modeling indicates that while the analytical multi-neutral-axis theory overestimates strain in the flexible chip structure under large bending deformation, it still precisely predicts the neutral axis location in the laminates. Therefore, we apply the configurational multi-neutral-axis design to experimentally demonstrate foldable photonic devices with extraordinary mechanical robustness that can sustain repeated bending at 0.25 mm bending radius. The fabricated TiO_2_ resonator devices exhibit an intrinsic Q-factor of 20,000. Low cytotoxicity of the sol-gel TiO_2_ material is confirmed through cell proliferation tests, making it a promising platform for biophotonic applications.

## Methods

### Sol-gel synthesis process

To prepare the sol, Titanium (IV) oxysulfate-sulfuric acid hydrate (TiOSO_4_∙xH_2_SO_4_∙xH_2_O) (Sigma-Aldrich, 99.99%) was first dissolved in distilled water at room temperature to form 0.05 mol/L TiOSO_4_ solution. The solution was then precipitated by adding ammonia solution (NH_3_·H_2_O, 3 mol/L) drop by drop during magnetic stirring until the pH value of the solution increased to 7–8. White titanium hydroxide (Ti(OH)_4_) powders precipitated in the solution and were subsequently filtered. To fully remove the NH_4_^+^ and SO_4_^2−^ ions, the titanium hydroxide precipitate was rinsed four times in DI water prior to re-dispersing the precipitates in DI water. Hydrogen peroxide (H_2_O_2_, 30%) was then added slowly to the suspension solution to obtain a yellow, translucent peroxo titanic acid (PTA, [Ti(O_2_)(OH)_2_]) complex sol^35^,^41^. The sol was stirred continuously for 6 to 10 hours to complete the gelation process. Lastly, the PTA sol was filtered and spin coated onto target substrates to form amorphous TiO_2_ thin films.

### TiO_2_ thin film characterization

We used infrared spectroscopy to monitor water and NH_3_ removal in annealed films. Infrared transmission spectra of the films were measured with a Perkin-Elmer Spectrum 100 series spectrometer with a Universal diamond ATR attachment. The TiO_2_ thin film thickness and refractive index at 1550 nm were calculated from the transmittance recorded by a Perkin-Elmer 1050 UV-Vis spectrophotometer. The index also matched well with the dispersion diagram fitted from the ellipsometry data collected at an angle of incidence of 70° in the range of 300–1600 nm (Horiba Jobin Yvon UVISEL NIR ellipsometer). The surface and cross-section images of the TiO_2_ thin film were taken on a JSM-7400 F (JEOL, Inc.) scanning electron microscope (SEM). Surface roughness was also measured through atomic force microscopy (AFM) on a Dimension 3100 (Digital Instruments, Inc.) microscope. Silicon AFM probes (Tap 150-G from Budget Sensors, Inc) with a force constant of 5 N/m and a resonant frequency of 150 KHz were used.

### Optical measurement

The transmission spectra of the TiO_2_ resonators and waveguides were measured by a tunable laser (Agilent Technologies, Model 81682 A) through tapered lens-tip fibers (Nanonics Inc.) end-fire coupling method as shown in Supporting information Figure S1. Figure 4a indicates the optical images of the fiber coupled to the end facet of the flexible waveguides delaminated from the handler substrate. TE-polarized light was used during the whole optical measurement. Figure 4b presents a far-field image of 1550 nm optical output from the measured waveguide.

### Cytotoxicity

For the cytocompatibility study, human bone marrow derived hMSCs (passage 6, Lonza, Walkersville, MD) were cultured using MSC maintenance medium (Lonza) in a humidified incubator at 37 ^o^C and 5% CO_2_. Upon reaching 80–90% confluence, cells were lifted and centrifuged before re-suspension in maintenance media. One milliliter of suspended cells (at 2 × 10^4^ cells/mL) was pipetted into each well of a tissue-culture treated 12-well plate (Corning). After allowing cells to attach for 2 hours, cell culture inserts (Millipore, 0.4 μm pore size) were added. Following a two day initial culture in the 12-well plates, the sample substrates were added inside each cell culture insert. The media was refreshed every other day, adding 800 μL to the well and 200 μL inside the cell culture inserts to ensure proper perfusion. On days 0, 2, 4, 6 and 8 a PrestoBlue (Life Technologies) cell viability assay was performed according to the manufacturer’s protocol. Briefly, following each culture period, cells were washed with sterile PBS before 1 mL of PrestoBlue viability reagent, diluted in maintenance media at a 1:9 ratio, was added to each well. Following a 30 minute incubation at 37 ^o^C, three 100 μL aliquots from each well were transferred to a 96-well plate for fluorescence measurement at 570/610 nm using a microplate reader. Statistical significance was determined by analysis of variance (ANOVA) with Tukey-Kramer post-hoc analysis.

For direct contact imaging, fifty microliters of hMSCs (at 4 × 10^5^ cells/mL) suspended in maintenance media were carefully pipetted onto each sample chip in a 24-well plate (Corning). Each chip had a surface area of approximately 0.36 cm^2^. After allowing cells to adhere to the surfaces for two hours, 400 μL of maintenance media was added to each well. Cells were cultured for 10 days, changing media every 3 days. Following the 10^th^ day of culture, cells were washed with PBS and a live/dead stain (Invitrogen) was added consisting of 5 μM SYTO13 and 0.75 μM propidium iodide in PBS. After a 15 minute incubation at 37 ^o^C, chips were transferred to two-well Nunc chamber slides (Thermo Scientific) for imaging on a Zeiss LSM 710 confocal microscope.

## Additional Information

**How to cite this article**: Li, L. *et al.* Foldable and Cytocompatible Sol-gel TiO_2_ Photonics. *Sci. Rep.* **5**, 13832; doi: 10.1038/srep13832 (2015).

## Supplementary Material

Supplementary Information
