# Supplementary material for: Foldable and Cytocompatible Sol-gel TiO2 Photonics
Source: Sci Rep. 2015 Sep 7;5:13832. doi: 10.1038/srep13832 (PMC4561379; doi:10.1038/srep13832)
Supplement: Supplementary Information [file srep13832-s1.pdf]

Additional information

Foldable and Cytocompatible Sol-gel TiO_2 Photonics

Lan Li[1], Ping Zhang[2], Wei-Ming Wang[3], Hongtao Lin[1], Aidan B. Zerdoum[4], Sarah J. Geiger[1], Yangchen Liu[1], Nicholas Xiao[1], Yi Zou[1], Okechukwu Ogbuu[1], Qingyang Du[1], Xinqiao Jia[1,4], Jingjing Li[3], and Juejun Hu[1,5]*

[1]University of Delaware, Department of Materials Science & Engineering, Newark, Delaware 19716, USA
[2]Tianjin University, School of Electronic and Information Engineering, Tianjin 300072, China
[3]University of Hawaii at Manoa, Department of Mechanical Engineering, Honolulu, Hawaii 96822, USA
[4]University of Delaware, Biomedical Engineering Program, Newark, Delaware 19716, USA
[5]Massachusetts Institute of Technology, Department of Materials Science & Engineering, Cambridge, Massachusetts 02139, USA
*E-mail: hujuejun@mit.edu

1. Optical transmission measurement setup

Figure S1 schematically illustrates the optical transmission measurement setup used to characterize the photonic devices. The fabricated flexible TiO_2 chip was first delaminated from the rigid silicon handler substrate using Kapton tape before it was mounted onto the linear motion sample stages. Bending radius of the chip was controlled by the distance between the two stages and measured from the image using the imaging processing software ImageJ.

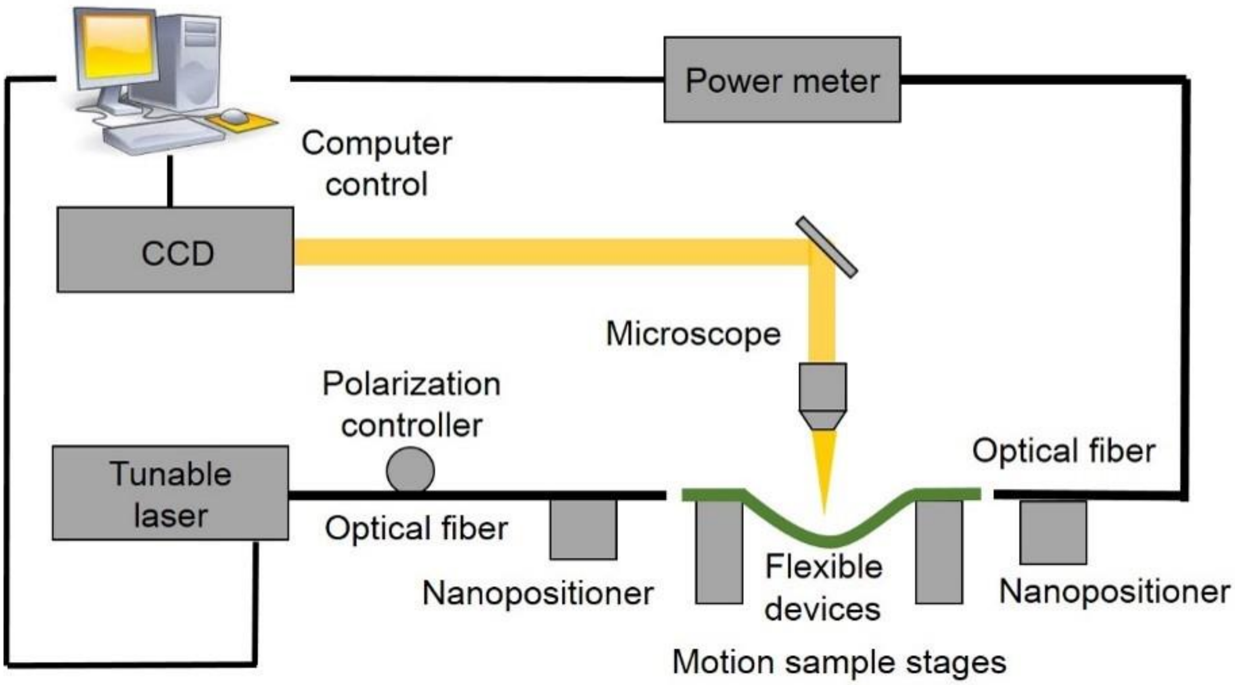

Figure S1. Schematic diagram of the testing setup.

2. The surface morphology of the TiO_2 rib waveguides by AFM

Figures S2a-b are AFM images showing the surface morphology of the TiO_2 waveguides without and with resist residues on the sidewalls. RMS roughness of (1.4 ± 0.3) nm was obtained by averaging 15 measurements on different waveguides. The low surface roughness incurs negligible scattering loss in the waveguides. However, we also observed the presence of discrete resist residue (a conclusion drawn from EDX composition micro-analysis) on some waveguide sections with large peak-to-peak roughness up to 40 nm. The measured roughness values indicate that the discrete resist residue defects are likely the dominant source of scattering loss.

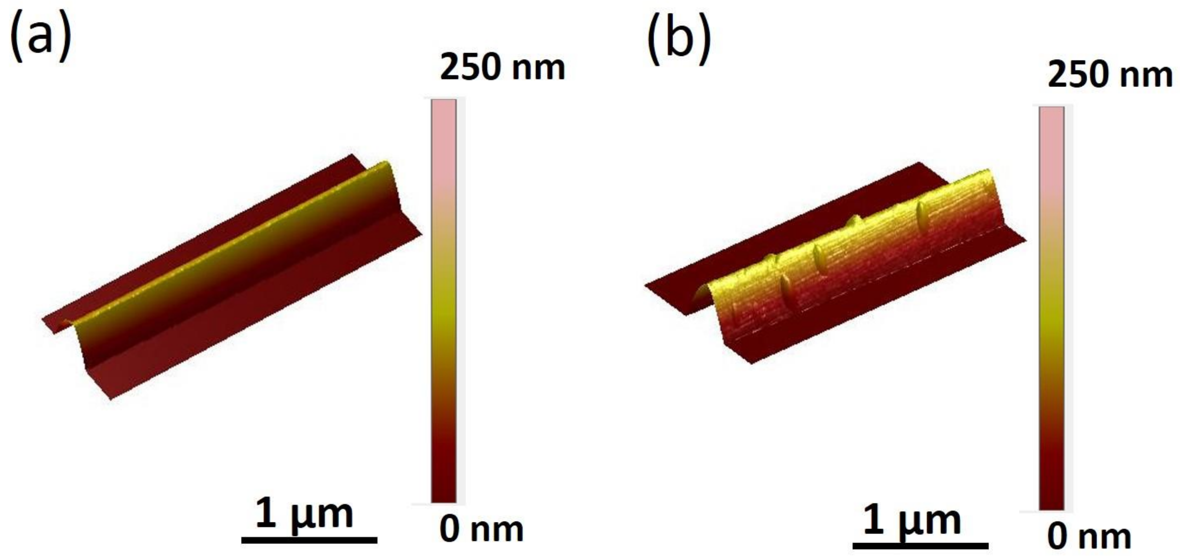

Figure S2. Surface morphology of TiO_2 rib waveguides by AFM: (a) a waveguide without resist residue; and (b) a waveguide with resist residue.

3. hMSC confluence on Day 8 of nonconformal contact cytotoxicity study

Phase contrast light microscope images presented in Figure S3 indicate a confluent monolayer of hMSCs on day 8 of the cytotoxicity test with the sensor materials suspended on top of the cell layer.

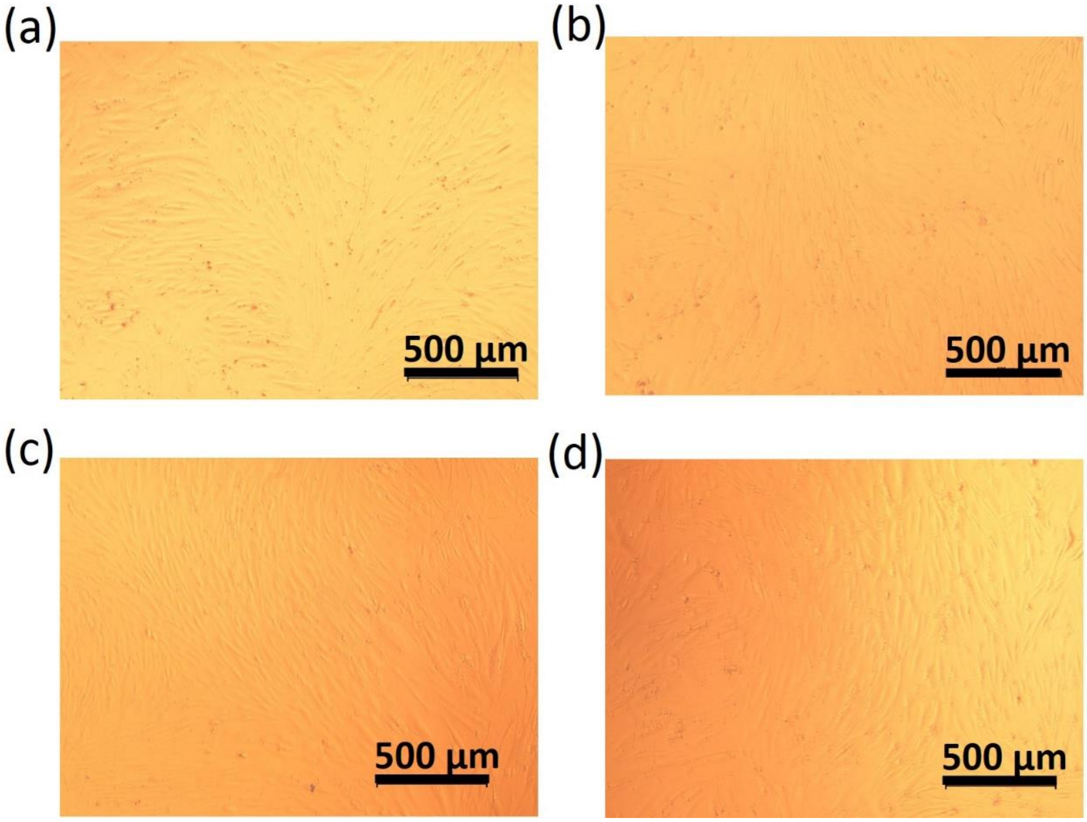

Figure S3. Evidence of hMSC confluence in wells containing: (a) Tissue culture plastic control, (b) Bare silicon wafer, (c) TiO_2 thin films on silicon, and (d) SU-8 on TiO_2 thin films on silicon.

4. FEM modeling parameter setting

In order to map strain distribution inside the bent flexible chip, eight-node solid elements were applied to all layers. Regarding the material models, the elastic material model [1] was applied to Polyimide, SU-8 and TiO_2. Silicone was treated as an incompressible hyperelastic solid and was characterized using the Blatz-Ko rubber model [1,2], where the Poisson's ratio was fixed to the default value of 0.463. The detailed material parameters used in FEM simulation are summarized in Table 1.

Table 1. Materials parameters used in FEM simulation

Materials	Young's Modulus (GPa)	Density (g/cc)	Poisson ratio
Polyimide<br>SU-8<br>TiO_2	2.5<br>2<br>147	1.42<br>1.12<br>3.82	0.34<br>0.22<br>0.50
Materials	Shear Modulus (MPa)	Density (g/cc)	Poisson ratio
Silicone	0.50	0.97	0.463

Using the FEM model, the effect of TiO_2 layer on deformation can be identified. Figure S4 compares the strain distributions along the centerline OO' in Figure 3a. The horizontal strain component (ε_x) in the polyimide and SU-8 layers are compared in Figure S4a-c for bending radii (R) of 1 mm, 0.85 mm, and 0.25 mm, respectively. In all cases, the TiO_2 layer decreased the compressive and tensile strains in both polyimide and SU-8 layers, and the decrease is most pronounced at R = 0.25 mm. Moreover, when the device was bent to the opposite direction (reverse bending shown in Figure S6), the presence of TiO_2 layer still decreases the strains in SU8 and polyimide layers in a similar manner. These results suggest that it is mandatory to include the TiO_2 layer (despite its small thickness compared to SU-8 and polyimide) in the FEM modeling to ensure accuracy of the result. The results presented in Figure 3 have incorporated the TiO_2 layer in the simulations.

Bending in the opposite direction (with the polyimide layer facing outwards) was also simulated, and the strain distributions are compared for both FEM and analytical models in Figure S5. At R = 1 mm and R = 0.85 mm, both models show good agreement. When R = 0.25 mm, the analytical model overestimates the strain. These results are consistent with our previous analysis (Figure 3).

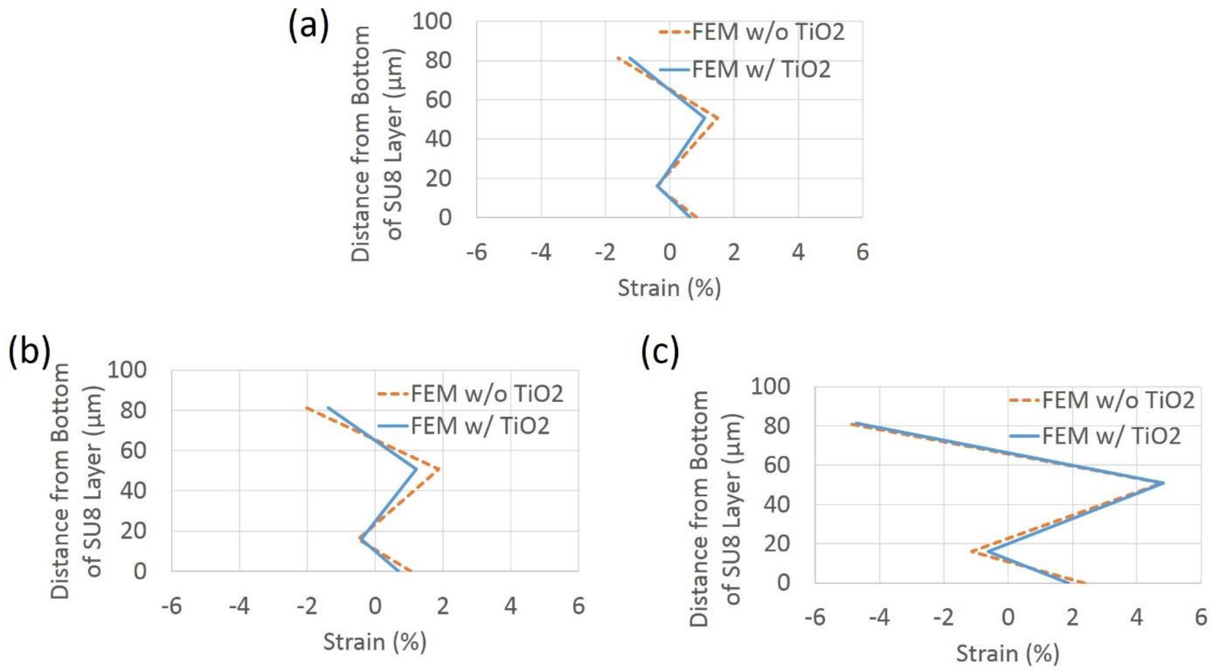

Figure S4. Comparison of ε_x along the OO’ axis for the cases with TiO_2 and without TiO_2: (a) R = 1 mm, (b) R = 0.85 mm, and (c) R = 0.25 mm.

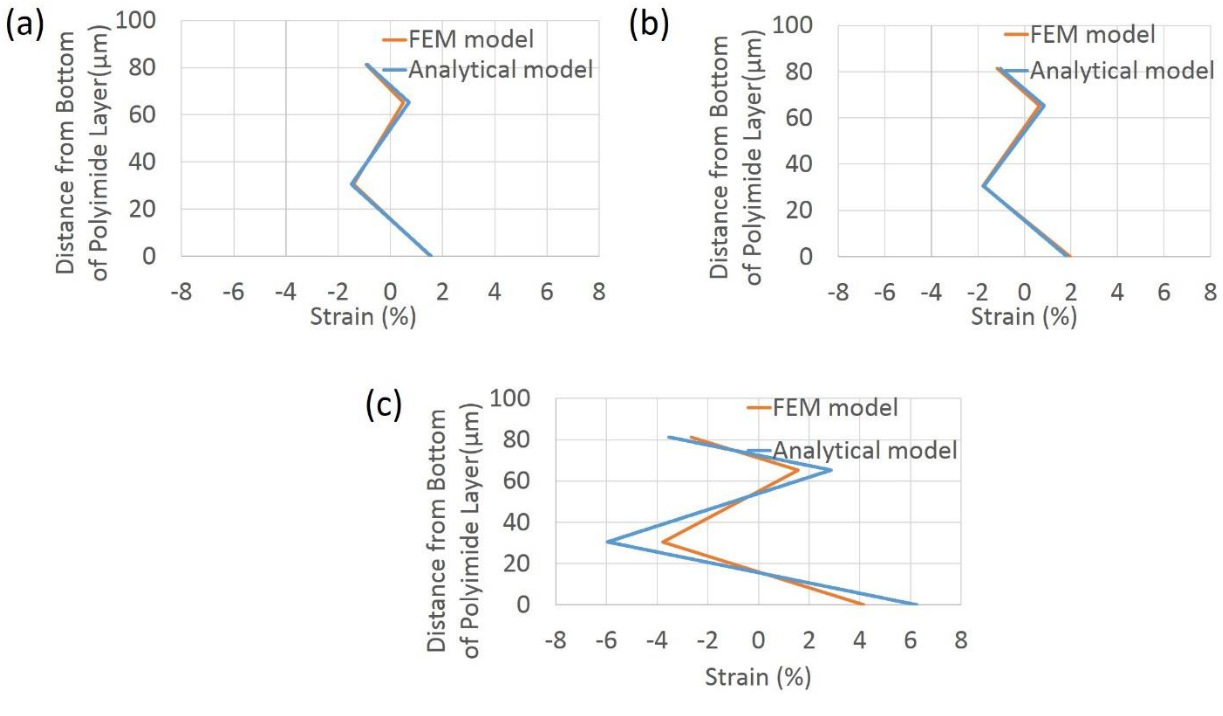

Figure S5. Strain ε_x along the structure's center axis OO' calculated using FEM and the analytical multi-neutral-axis model when the bending direction is reversed (reversed bending): (a) R = 1 mm, (b) R = 0.85 mm, and (c) R = 0.25 mm.

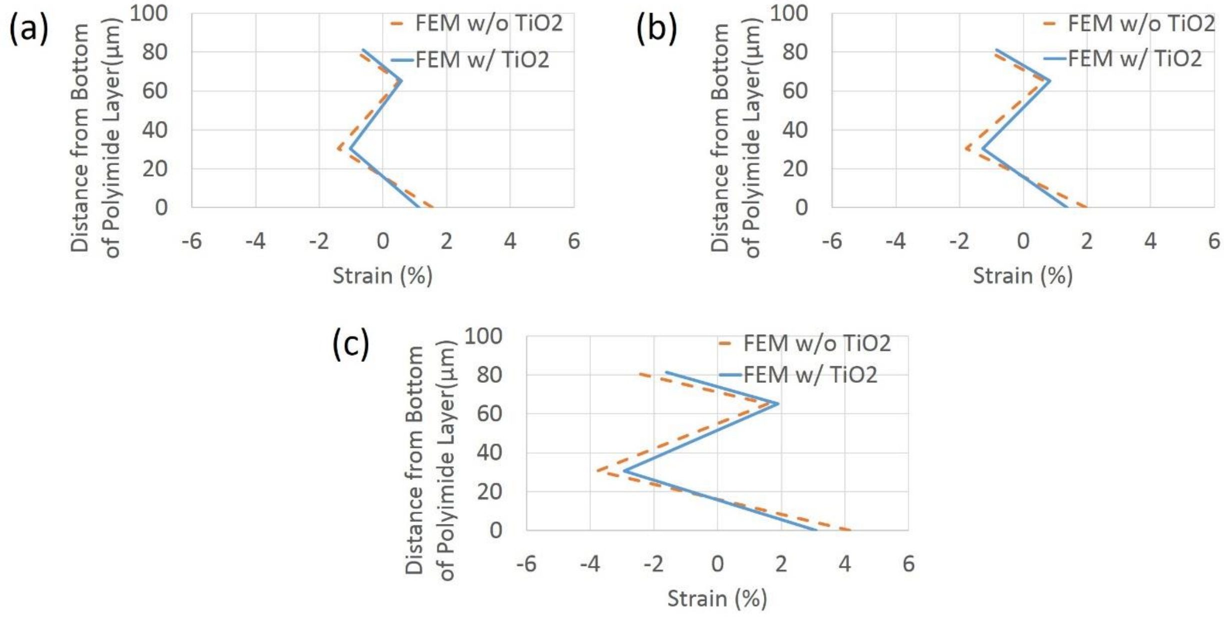

Figure S6. Comparison of ε_x along the OO' axis for the cases with TiO_2 and without TiO_2 (reversed bending): (a) R = 1 mm, (b) R = 0.85 mm, and (c) R = 0.25 mm.

5. Sputtering deposited TiO_2 thin films: a comparison

RF reactive sputtering method was also practiced to deposit TiO_2 thin films on both rigid and flexible substrates. A TiO_2 (99.9%, 2 inch in diameter, 0.125 inch in thickness, Plasmaterials Inc.) target was sputtered in an Ar:O_2 ambient (at flow rate ratios of 90:10 or 80:20) at a working pressure of 2.5 mTorr. Before deposition, the chamber was pumped down to a base pressure of less than 10^{-6} Torr. The TiO_2 target was pre-sputtered for 5 minutes to remove any possible surface contamination of the target prior to initiating deposition on the substrates. The deposition rate of the film was 38 nm/hour at an RF power of 190 W.

Figure S7a shows XRD patterns of TiO_2 thin films on glass slides deposited at different sputtering conditions. In general, increasing oxygen partial pressure or increasing sputtering time leads to higher crystallinity in the resulting films. Figure S7b gives surface top-view and cross-section images of the film deposited at 10% oxygen flow ratio and 190 W for 14 hours, where the crystal grains are clearly visible. We also observed severe polymer substrate damage during sputtering deposition due to oxygen plasma ashing. The substrate etching effect is evident from Figure S7c and S7d, which show the cross-section of a TiO_2 film sputtered deposited onto an SU-8 substrate

on which a trench is lithographically defined in NR9 photoresist (Futurrex Inc.). The SU-8 substrate maintains a smooth surface in the NR9 protected regions whereas significant SU-8 surface etching and roughening is observed inside the trench. Based on the results, we conclude that sputtering is not applicable to TiO_2 photonic fabrication on flexible substrates.

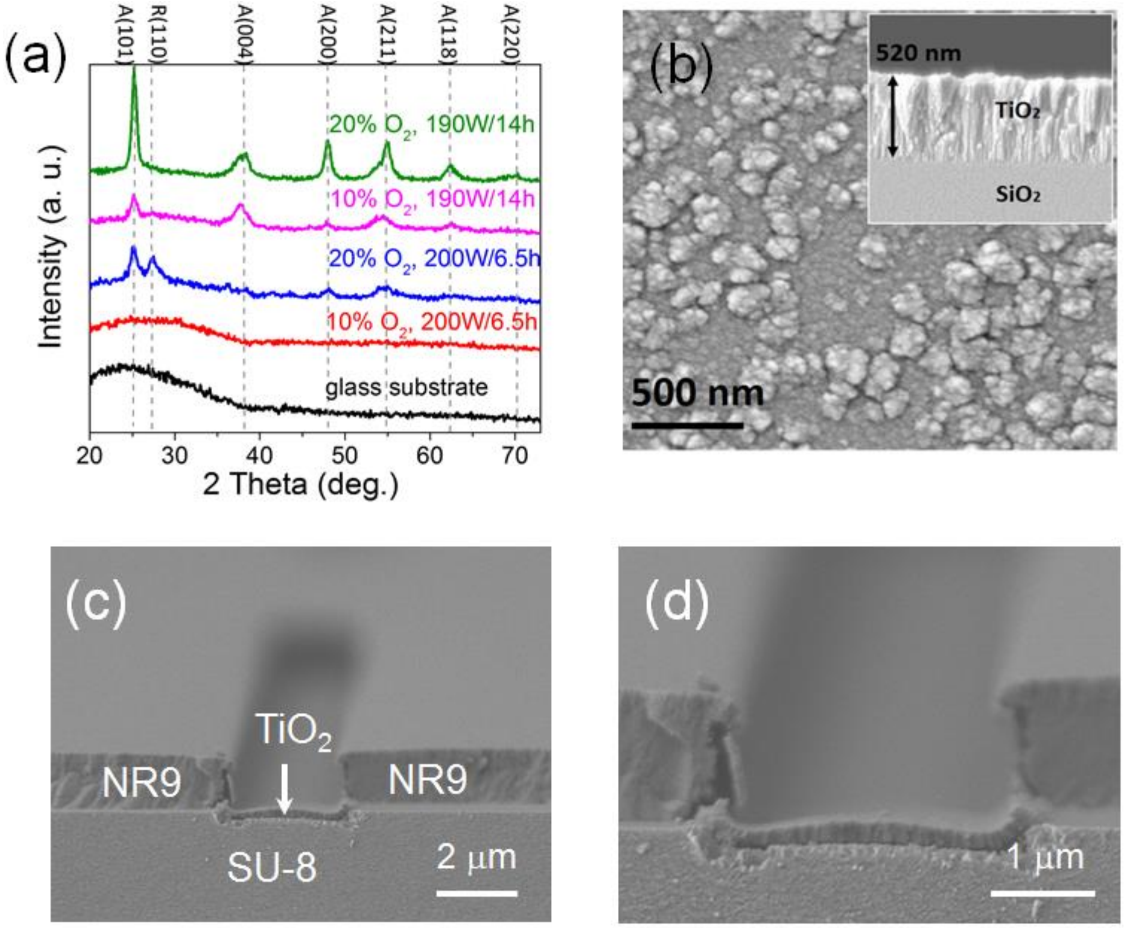

Figure S7. (a) X-ray diffraction spectra of TiO_2 thin films sputter deposited at different conditions. Diffraction peak positions of the anatase phase (labeled as "A") and the rutile phase (labeled as "R") quoted from JCPDS 84-1286 are also marked for comparison. (b) Top-view surface and cross-section SEM images of TiO_2 film sputtered at 10% oxygen flow ratio and 190 W for 14 hours. (c, d) SEM images of a sputtered TiO_2 film on an SU-8 substrate. A trench is lithographically defined in NR9 photoresist which serves as a resist mask. Substrate damage due to oxygen plasma is clearly visible in (d).

1. Hallquist, J. Ls-Dyna Theory Manual. http://www.lstc.com/pdf/ls dyna_theory_manual_2006.pdf (March, 2006)
2. Bondi, S. Capturing structural silicone non-linear behavior via the finite element method. in *Glass Performance Days* (Tampere, 2009).
